# DAB2IP: unifying cardiovascular pathogenesis and cardiovascular brain crosstalk

**DOI:** 10.3389/fcvm.2026.1660204

**Published:** 2026-02-03

**Authors:** Yufan Zhou, Peiwen Yang, Hanxun He, Hao Liu, Ping Ye, Jiahong Xia

**Affiliations:** 1 Department of Cardiovascular Surgery, Union Hospital, Tongji Medical College, Huazhong University of Science and Technology, Wuhan, China; 2 Department of Vascular Surgery, Traditional Chinese and Western Medicine Hospital of Wuhan, Tongji Medical College, Huazhong University of Science and Technology, Wuhan, China; 3 Department of Cardiology, Central Hospital of Wuhan, Tongji Medical College, Huazhong University of Science and Technology, Wuhan, China

**Keywords:** apoptosis, atherosclerosis, cardiovascular-brain crosstalk, cardiovascular diseases, DAB2IP, inflammation

## Abstract

Cardiovascular diseases and brain disorders collectively represent a major global health burden. Not only do they frequently occur as comorbidities, but their pathological mechanisms are also intricately linked through a complex network of cardiovascular-brain crosstalk. Cardiovascular diseases (CVDs) and neurological disorders together impose high global health burdens. The *DAB2IP* gene, initially characterized as a tumor suppressor, serves as a multifunctional regulator and performs roles in both the cardiovascular and neurological systems. In atherosclerosis (AS), DAB2IP suppresses the inflammation and apoptosis of endothelial cells (ECs) through the TNF signaling pathways, inhibits phenotypic switching of vascular smooth muscle cells (VSMCs) via the JAK-STAT and PI3K-Akt axes, and attenuates plaque angiogenesis via VEGF-related pathways. It is genetically associated with coronary artery disease (CAD), aortic aneurysm (AA), and aortic dissection (AD) risk, and mediates hemodynamic stress responses and glucose/lipid metabolic dysregulation in the vasculature. In the cardiovascular brain circuit (CBC), DAB2IP governs cortical neuron migration through the Rap1-integrin pathways, modulates the integrity of the blood-brain barrier (BBB) in Alzheimer's disease models via apoptosis-related signaling, and associates with arterial adventitial immune-neural remodeling. Newly developed DAB2IP-targeted strategies, including epigenetic modulators and engineered exosomal circRNA delivery systems, demonstrate preclinical potential but require rigorous validation in various models to assess long-term biosafety and organ-specific efficacy.

## 1 Introduction

Cardiovascular diseases (CVDs) and neurological disorders are the leading causes of morbidity and mortality worldwide. Not only do they frequently occur as comorbidities, but their pathological mechanisms are also intricately linked through a complex network of cardiovascular-brain crosstalk (1). CVDs encompass a broad spectrum of conditions characterised by structural and functional impairment of the heart and vasculature, driven by an intricate and multifactorial pathophysiological basis. Its pathogenesis is closely associated with canonical risk factors such as advanced age(>65 years), male sex, exposure to tobacco, hypertension, hyperlipidemia, insulin resistance, and obesity-related metabolic disorders (2). They are likewise established drivers of neurodegenerative pathologies (3). According to the 2025 Heart Disease and Stroke Statistics, drawing on the Global Burden of Disease study, CVDs were responsible for about 19.41 million deaths globally in 2021 (age-standardized mortality rate of 235.18 per 100,000 population), with a staggering global prevalence of 612.06 million cases (age-standardized prevalence rate of 7178.73 per 100,000), confirming their status as the dominant contributor to the global noncommunicable disease burden (4). Notably, the global burden of neurological disorders in 2021 was also substantial, with about 11.10 million deaths (age-standardised mortality rate of 39 per 100,000) and 3.4 billion prevalent cases (age-standardised prevalence rate of 41,204.1 per 100,000) (5). Moreover, recent studies report that individuals with high composite cardiovascular risk scores face a 1.67-fold increased risk of Alzheimer's disease and a 1.53-fold increased risk of vascular dementia compared to those with low scores (6).

As early as the 19th century, Claude Bernard posited that the brain and the heart are not isolated from each other; instead, they form a complex bidirectional regulatory network through neural, humoral, immune, and other pathways, giving rise to the concept of the cardio-cerebral circuit (CBC). As a physiological link between the cardiovascular and nervous systems, the CBC comprises the heart-brain circuit (HBC) and the artery-brain circuit (ABC), which respectively physiological connection between the heart and the brain, and between the arterial system and the brain (1). In recent years, key neurophysiological evidence has further advanced this field, including the first whole-brain dynamic spatiotemporal mapping of human brain responses to cardiac rhythms (7). As a cutting-edge area of research, the CBC continues to attract growing attention.

Nevertheless, the key target proteins linking cardiovascular and neurological pathologies remain elusive. This review centers on the *DAB2IP* gene encodes the disabled homolog 2-interacting protein (DAB2IP), which was originally identified as a tumor suppressor and was later found to serve as a key regulator in both the cardiovascular and neurological systems. Owing to its specific interaction with apoptosis signal-regulating kinase 1 (ASK1), this protein is also designated ASK1-interacting protein 1 (AIP1). The expression of DAB2IP is epigenetically silenced in various types of cancers, including prostate, breast, ovarian, and colorectal cancers, through regulatory mechanisms involving histone modifications mediated by Polycomb repressive complex 2 and Enhancer of Zeste Homolog 2 (EZH2), Smurf1-mediated ubiquitin-proteasomal degradation, and the action of mutant p53 (8).

At the structural level, DAB2IP exhibits significant polymorphisms, encoding multiple protein variants with distinct N- terminal and C-terminal regions (five isoforms have been identified in human populations), all sharing six conserved domains: a pleckstrin homology-like (PH) domain, protein kinase C-conserved region 2 (C2), a Ras-GTPase-activating protein (GAP) domain, a C-terminal PERIOD-like (PER) domain, a proline-rich (PR) region, and a leucine-zipper motif—; these structural features are essential for its scaffolding function in various signaling cascades (9) (Central Illustration).

**Central Illustration:**
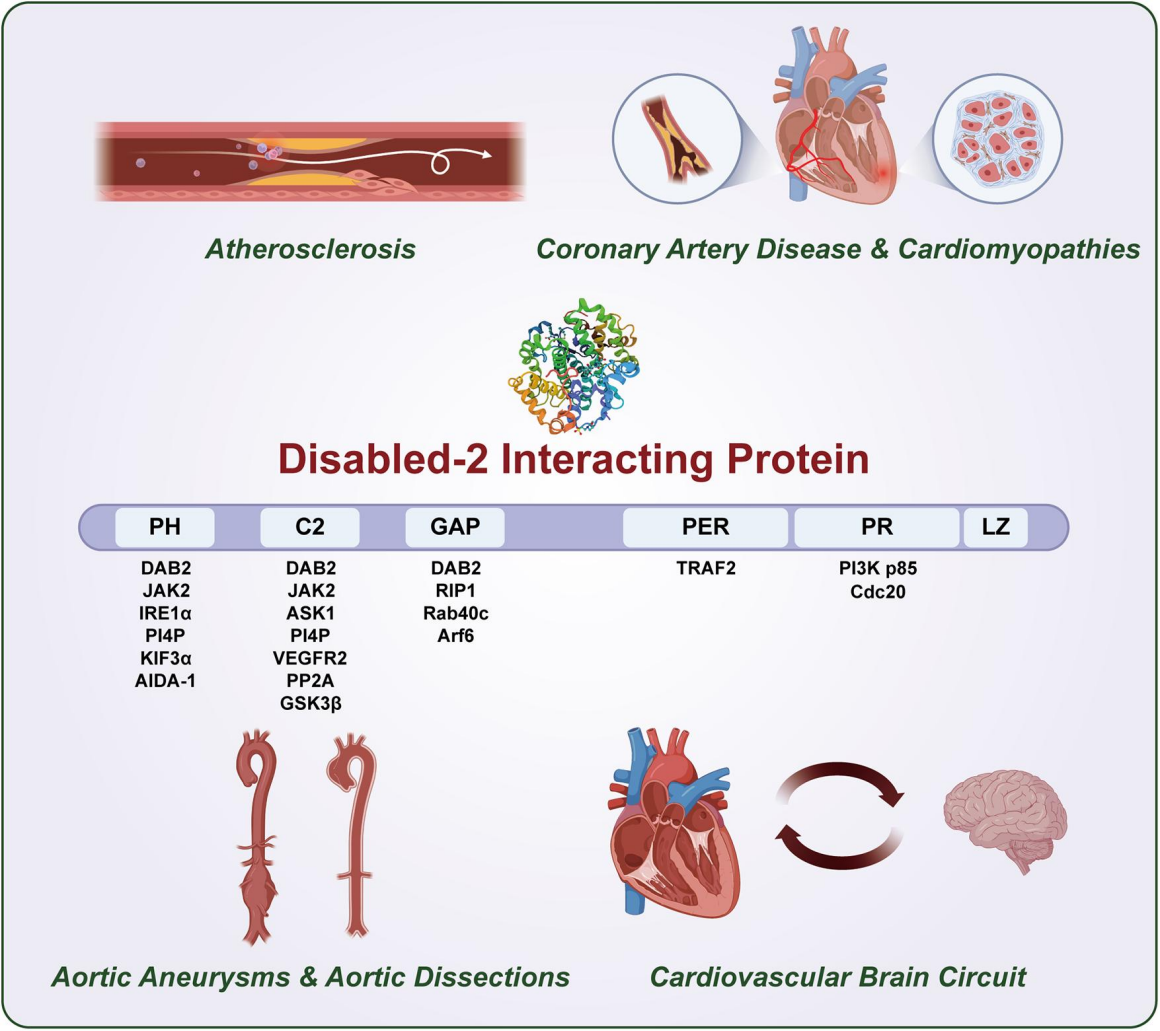
DAB2IP is central integrator of cardiovascular disease spectrum and cardiovascular brain circuit.

Although researchers have extensively characterized DAB2IP in oncology, its functional significance in cardiovascular and neurological systems remains poorly investigated. In this review, we comprehensively described the key regulatory mechanisms of DAB2IP as a multifunctional signaling hub in various cardiovascular pathologies, including AS, CAD, AA, and AD (10–13). We also assessed the plausible roles of DAB2IP in CBC as an informed speculation, based on its established molecular functions and signaling mechanisms in various physiological contexts (1). Additionally, we critically evaluated its potential as a pleiotropic modulator for therapeutic interventions targeting both CVDs and cardio-cerebral comorbidities (Central Illustration).

## 2 DAB2IP suppresses atherosclerosis and transplant arteriosclerosis

**Figure 1:**
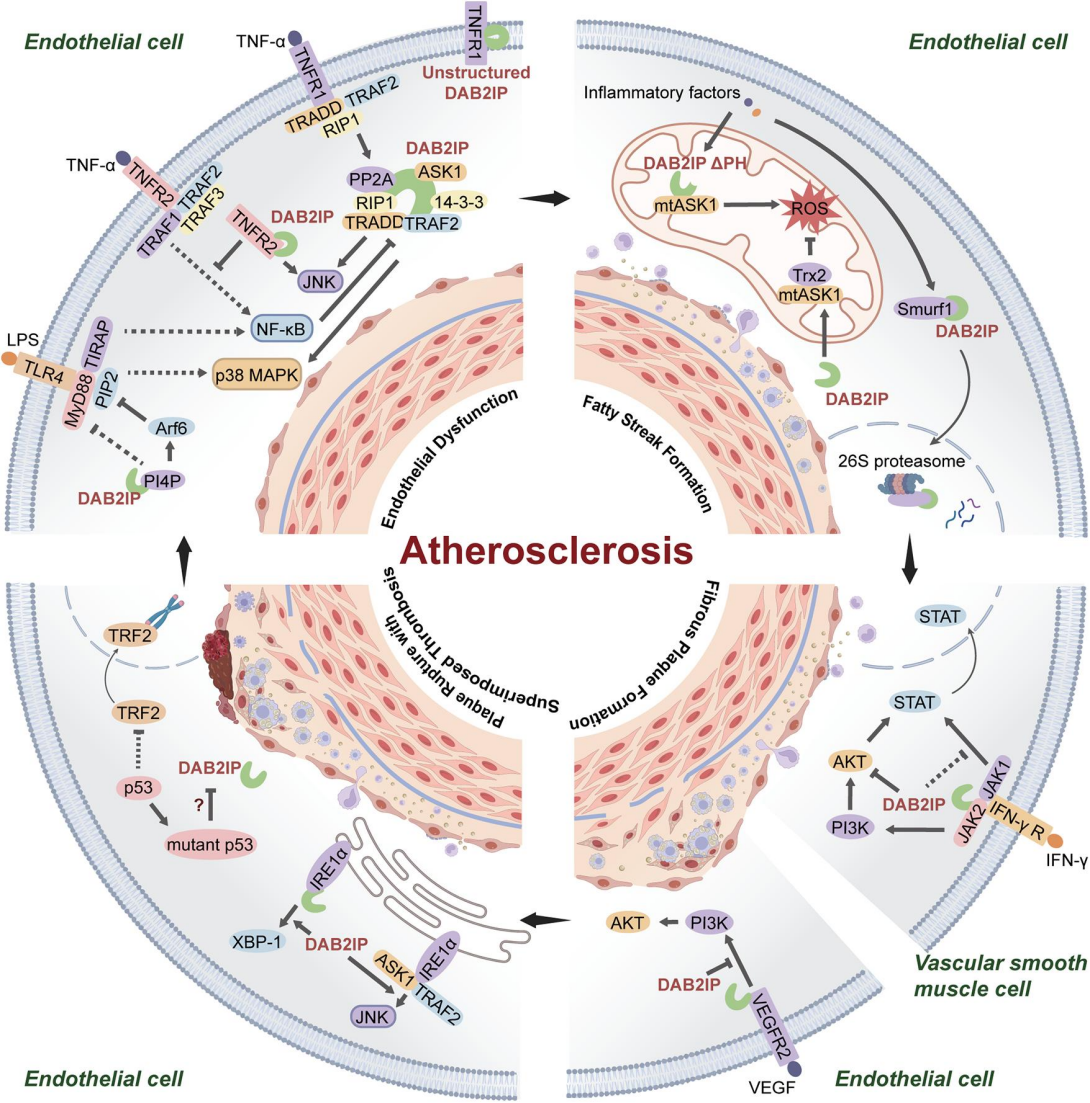
DAB2IP-mediated signaling pathways in atherosclerotic progression. **(**1) DAB2IP inhibits TNF-α-mediated inflammatory pathway formation and TLR4-TIRAP-MyD88 complex assembly, resulting in endothelial dysfunction; (2) Fatty streak phase inflammatory stress triggers normal DAB2IP degradation and activation of aberrant mitochondrial DAB2IP (lacking PH domain), promoting ROS production; (3) DAB2IP inhibits IFN-γ-induced PI3K/AKT and JAK2/STAT1/3 signaling in VSMCs and blocks VEGFR2/PI3K p85 complex formation in ECs during fibrous plaque stage, suppressing excessive apoptosis; (4) DAB2IP drives IRE1α/XBP1 ER stress and IRE1-TRAF2/JNK activation in unstable plaques; role of p53 senescence in DAB2IP regulation undetermined.

DAB2IP suppresses atherosclerosis. A study published in 2013 reported atherosclerotic lesions were significantly exacerbated in *ApoE*^−/−^*Dab2ip*^−/−^ mice compared to the *ApoE*^−/−^ mice, characterized by an increase in the size and number of plaques, particularly in the aortic arch, thoracic aorta, and abdominal aorta, with severe coronary lesions (14). This effect stems from the role of DAB2IP as a pleiotropic signaling hub that protects vessels through coordinated regulation of several pathophysiological axes: inflammatory responses, oxidative/endoplasmic reticulum stress, apoptosis/pyroptosis, and vascular remodeling (Figure 1).

### 2.1 DAB2IP maintains endothelial homeostasis

Endothelial homeostasis acts as the primary barrier against atherosclerotic initiation. Its disruption precipitates heightened vascular permeability, diminished nitric oxide bioavailability, and aberrant endothelial cell activation. This activated phenotype facilitates the recruitment and adhesion of immune cells, thereby initiating early vascular dysfunction (15). DAB2IP serves as a central protector of endothelial equilibrium within this defense system by regulating key processes such as inflammatory resolution, apoptotic clearance, and immune cell recruitment. This underscores its strategic position at the role in preventing the incipient molecular events that catalyze plaque formation.

DAB2IP is a strong regulator of inflammation and participates in the inflammation associated with atherosclerotic lesions by regulating tumor necrosis factor (TNF)-mediated nuclear factor kappa-light-chain-enhancer of activated B cells (NF-κB) signaling and mitogen-activated protein kinase (MAPK) cascade activation in ECs. In the resting state, DAB2IP maintains a closed conformation through its PR domain. Following TNF stimulation, tumor necrosis factor receptor 1 (TNFR1) undergoes exposure to the death domain, which enables recruitment of tumor necrosis factor receptor-associated death domain protein (TRADD). Simultaneously, DAB2IP rapidly dissociates from TNFR1 within 15 min and is subsequently recruited by TRADD into an intermediate signaling complex distinct from canonical complexes I/II. Receptor-interacting protein kinase 1 (RIP1) then interacts via its intermediate domain with the GAP domain of DAB2IP, triggering phosphorylation at Ser604 within the C-terminal region of DAB2IP. This phosphorylation event induces a conformational opening of DAB2IP, which is required for subsequently recruiting TRADD and assembling the TRADD-DAB2IP-RIP1-TRAF2 intermediate complex. The conformation opening enables the PER domain (aa 591–719) of DAB2IP to interact with the RING finger domain of TNF receptor associated factor 2 (TRAF2), effectively redirecting the function of TRAF2 from NF-κB activation toward the mitogen-activated protein kinase 3 (MAP3K) signaling cascades. This functional switch is further enhanced through the autophosphorylation of RIP1. Besides its indirect modulation of TRAF2 activity, DAB2IP directly activates apoptosis signal-regulating kinase 1 (ASK1) via autophosphorylation of its C2 domain at Thr845, thereby initiating downstream Jun N-terminal kinase (JNK) and p38 mitogen (16).

Besides playing a role in TNF signaling, DAB2IP strongly suppresses MyD88-dependent early inflammatory responses via the TLR4 signaling pathway. DAB2IP binds phosphatidylinositol 4-phosphate via its PH and C2 domains and activates Arf6 through its GAP domain. These dual mechanisms synergistically decrease the production of phosphatidylinositol 4,5-bisphosphate and disrupt phosphatidylinositol 4,5-bisphosphate production-dependent assembly of the TLR4-TIRAP-MyD88 complex, thereby suppressing activation of the downstream NF-κB and MAPK signaling pathways (17). TLR4 signaling functions during the activation of endothelial injury, and is also expressed on macrophages and smooth muscle cells; however, relevant studies are lacking (18).

DAB2IP promotes apoptosis through the activation of ASK1 via complex formation, and the activation of JNK via the engagement of internalized TNFR2 in ECs. DAB2IP was initially identified as an ASK1-interacting protein through a yeast two-hybrid screen. DAB2IP promote the dissociation of the ASK1 inhibitory protein 14-3-3 and recruit protein phosphatase 2A (PP2A) to the ASK1 complex. This conclusion is supported by siRNA knockdown, mutant expression, inhibitor treatment, and co-immunoprecipitation experiments. Further research revealed that Ser604 on DAB2IP is a 14-3-3 binding site, regulated by the upstream kinases RIP1 and Homeodomain Interacting Protein Kinase 1 (HIPK1). The function of this site was confirmed through bioinformatic prediction, site-directed mutagenesis, glutathione S transferase pull-down assay, and phospho-specific antibody detection. Gain- and loss-of-function experiments established RIP1 as the direct kinase phosphorylating Ser604, while HIPK1 was identified via a yeast two-hybrid screen using DAB2IP as bait. Cellular localization and a SUMOylation-defective mutant demonstrated that TNF-induced desumoylation and cytoplasmic translocation of HIPK1 cooperate with the DAB2IP–ASK1 complex to promote the dissociation of thioredoxin and 14-3-3 from ASK1, thereby driving apoptosis (10). A parallel apoptotic pathway involves tumor necrosis factor receptor 2 (TNFR2). In 2012, Wang Min's team demonstrated TNFR2 on the plasma membrane activates the NF-κB pathway via its TRAF2 binding site through domain deletion, subcellular localization analyses, and protein-protein interaction assays. However, functional domain screening and apoptosis experiments collectively showed that internalized TNFR2 can directly bind DAB2IP through its domain III, thereby activating JNK and inducing endothelial cell apoptosis. Based on the observation that the TNFR2-59 mutant exhibits stronger JNK activity, the authors speculate that when the TRAF2 binding site is present, TNFR2-activated inhibitor of kappa B kinase indirectly inhibits JNK pro-apoptotic signaling by preventing DAB2IP from associating with the TRAF2/RIPK1 complex (19).

DAB2IP modulates the progression of immune responses by regulating the activity of ECs rather than exerting effects in macrophages. The *ApoE*^−/−^*Dab2ip*^−/−^ mice presented increased expression of the NF-κB-dependent EC adhesion molecule ICAM-1 and pro-inflammatory genes (*TNF*, *IL-6*, and *IL-12*). Moreover, the levels of macrophage recruiting chemokines (monocyte chemoattractant protein-1, regulated on activation normal T expressed and secreted, and C-X3-C motif chemokine ligand 1) were significantly higher at lesion sites, accompanied by an increase in the infiltration of CD68 + macrophages. Experiments designed to assess the direct effect of DAB2IP on macrophages revealed that the systemic counts of circulating monocytes and CD4^+^ T cell subsets (including Th1 and Treg populations) in these double-knockout mice remained unchanged. Moreover, adoptive transfer of *ApoE*^−/−^*Dab2ip*^−/−^ mice macrophages into *ApoE*^−/−^ mice failed to enhance inflammation or AS. These findings indicate that DAB2IP does not directly affect monocytes/macrophages but instead exacerbates endothelial injury by promoting chemokine release from activated endothelium, which recruits macrophages (14).

To summarize, DAB2IP orchestrates endothelial homeostasis at the inception phase of AS through the tripartite regulation of inflammatory resolution, apoptotic clearance, and immune response. Its dysfunction accelerates endothelial barrier failure, creating a permissive niche for the infiltration of monocytes and the retention of subendothelial lipids.

### 2.2 DAB2IP orchestrates fatty streak development through oxidative stress and lipid accumulation

The fatty streak stage of AS, characterized by abundant foam cell formation, and extracellular lipids, represents a reversible phase strongly driven by oxidative stress. In this early lesion, the oxidation of low density lipoprotein (LDL) to oxidized low-density lipoprotein (ox-LDL) serves as the primary molecular trigger for foam cell genesis (20).

In AS, a decrease in the levels of normal DAB2IP and an increase in the levels of DAB2IP lacking the PH domain (DAB2IP-ΔPH) promote mitochondrial the production of reactive oxygen species (ROS). Under physiological conditions, replication timing regulatory factor 1 binds to chromatin and recruits H3K9 methyltransferases, producing a compact H3K9me3 chromatin structure that effectively suppresses the transcription of DAB2IP-ΔPH. However, in human aortic ECs from AS patients, normal DAB2IP is ubiquitinated by Smurf1 and transported to the 26S proteasome for degradation, while DAB2IP-ΔPH, which is localized to mitochondria, is substantially released. This shift leads to dual detrimental effects: the anti-inflammatory protective effect of normal DAB2IP is compromised, while DAB2IP-ΔPH promotes TNF-α-induced mitochondrial ROS production, ultimately significantly aggravating oxidative stress damage (21).

The anti-atherogenic effects of DAB2IP are not mediated through the regulation of systemic lipoprotein levels, but rather through the local control of lipid accumulation within foam cells. Evidence for this view comes from prior genome-wide association study (GWAS) revealed no significant associations between *DAB2IP* gene and other lipoproteins (11, 12). Additionally, despite strong historical linkage disequilibrium (D′ = 0.709) between the *LPA* rs3798220 [C] variant, associated with ox-LDL variations in systemic AS and other CVDs, and *DAB2IP* rs7025486 [A], their functional interplay remains minimal (*r*^2^ = 0.002). These phenotypes are more likely mediated through independent biological mechanisms (22) (Table 1). The core mechanism of DAB2IP likely involves the regulation of intracellular lipid homeostasis. *In vitro* evidence indicates that DAB2IP acts as a Ras GTPase-activating protein for RAB40C, suppressing RAB40C-mediated lipid droplet overaccumulation in hepatorenal cell lines (28). In the context of atherosclerosis, this function may be crucial, as the phagocytosis of oxidized low-density lipoprotein by macrophages via scavenger receptors such as SR-A and CD36 and their subsequent differentiation into foam cells represent a central step in lesion formation. However, whether this regulatory function extends to VSMCs or macrophages requires further validation. Given that ROS promotes ox-LDL generation and foam cell formation, DAB2IP may influence ROS production.

**Table 1:** Association of DAB2IP single-nucleotide polymorphisms.

SNP	Phenotype	OR/ HR (95% CI)	*p*-value	Ref.
Cardiovascular diseases				
*DAB2IP* rs7025486(A)	CAD	1.10 (1.06–1.14)	3.2 × 10^−6^	(11)
*DAB2IP* rs7025486[A]	Early-onset CAD	3.43 (0.92–12.53)	0.08	(23)
*DAB2IP* rs7025486(A)	CAD	*NA*	0.530	(24)
*DAB2IP* rs587936(C)	CAD survival outcomes	0.65 (0.51–0.83)	2.3 × 10^−5^	(25)
*DAB2IP* rs885150(G)	CAD	0.97 (0.95–0.98)	7.8 × 10^−10^	(26)
*DAB2IP* rs7025486(A)	PMI	1.18 (1.09–1.27)	3.1 × 10^−5^	(12)
*DAB2IP* rs7025486(A)	AAA	1.21 (1.11–1.32)	4.6 × 10^−10^	(12)
*DAB2IP* rs7025486(A)	AAA	0.46 (0.22–0.98)	0.040	(22)
Cardiocerebrovascular diseases				
*DAB2IP* rs7025486(A)	IA	1.02 (0.89–1.17)	0.77	(12)
*DAB2IP* rs7025486(A)	IS	1.07 (0.99–1.15)	0.097	(12)
*DAB2IP* rs7025486(A)	LVD	1.05 (0.86–1.27)	0.63	(12)
*DAB2IP* rs7025486(A)	CS	1.09 (0.94–1.27)	0.26	(12)
*DAB2IP* rs7025486(A)	SVC	0.98 (0.81–1.20)	0.89	(12)
Nervous diseases				
*DAB2IP* rs10818576(G)	Alzheimer's disease	0.94	2.4 × 10^−4^	(27)

AAA, abdominal aortic aneurysms; CAD, coronary artery disease; CS, cardiogenic stroke; HR, hazard ratio; IA, intracranial aneurysm; IS, ischemic stroke; LVD, large vessel disease; NA, not available; OR, odds ratio; PMI, premature myocardial infarction; SNP, single nucleotide polymorphisms; SVC, small vessel disease.

Hypoxia exacerbates this process through an independent mechanism. A study found that hypoxia-induced hypoxia inducible factor-1 alpha (HIF-1α) upregulates miR-1307-3p, which suppresses the expression of DAB2IP. This suppression triggers the activation of the AKT/mTOR pathway, further stabilizing HIF-1α and establishing a pathological feedback loop (29). Persistently activated HIF-1 strongly upregulates the expression of the scavenger receptor CD36, promoting lipid phagocytosis by macrophages while simultaneously regulating phenotypic switching of VSMCs (30). These mechanisms collectively drive the progression of atherosclerotic lesions.

Targeting the function of DAB2IP during the reversible fatty streak stage may provide a strategic intervention window. By modulating RAB40C-dependent lipid droplet homeostasis and suppressing mitochondrial ROS generation, this approach can simultaneously inhibit foam cell formation and oxidative stress damage, thereby offering a promising early intervention strategy to arrest the progression of atherosclerosis.

### 2.3 DAB2IP antagonizes VSMC phenotype switching and pathological angiogenesis

During the progression of atherosclerotic plaques, VSMCs migrate into the intima and adopt a synthetic phenotype, proliferating and secreting extracellular matrix (ECM) to form a fibrous cap that overlies the lipid-rich necrotic core. At this stage, the migration and proliferation of VSMCs, along with intraplaque neovascularization, are two key determinants of plaque stability and clinical prognosis (20).

DAB2IP specifically inhibits interferon-gamma (IFN)-γ-mediated abnormal migration, proliferation, and the inflammatory response in VSMCs. Using a mouse model of graft AS, a study conducted in 2011 demonstrated that DAB2IP suppresses abnormal migration and proliferation of VSMCs by inhibiting the activation of JAK2/STAT1/3 and PI3K/Akt induced by IFN-γ (31). Subsequent mechanistic studies demonstrated that IFN-γ induces the binding of DAB2IP to janus kinase 2 (JAK2), an interaction confirmed by co-immunoprecipitation and time-course analysis. Upon binding, DAB2IP suppresses JAK2 autophosphorylation, thereby disrupting the JAK-STAT signaling cascade. In DAB2IP-deficient cells, Western blot and immunofluorescence analyses revealed enhanced phosphorylation and nuclear translocation of signal transducer and activator of transcription 1/3 (STAT1/3), indicating that DAB2IP inhibits the transcription of STAT-dependent downstream genes. Furthermore, DAB2IP negatively regulates Akt activation. Experiments showed that knockdown of DAB2IP elevated IFN-γ-induced phosphorylation of both protein kinase B (Akt) and mammalian target of rapamycin (mTOR), an effect reversible by JAK2 inhibition, suggesting that DAB2IP restrains the Akt-mTOR pathway indirectly via suppression of JAK2. Consequently, DAB2IP reduces the synthesis of proliferation-associated proteins and ultimately attenuates pathological VSMC activation (32). Notably, experimental evidence demonstrates that DAB2IP linearly inhibits both the JAK2/STAT and PI3K/Akt pathways, an integrated model further proposes the existence of significant crosstalk between these pathways, potentially forming a feed-forward loop that amplifies the dysfunction of VSMCs (33). This model posits that by simultaneously targeting two signaling pathways, DAB2IP is positioned to disrupt their cross-activation, thereby effectively restraining the synthetic phenotype of VSMCs. The role of DAB2IP in suppressing the inflammatory response is directly evidenced by upregulating the expression of pro-inflammatory chemokines, including C-X-C motif chemokine ligand 9 (CXCL9), CXCL10, and CXCL11, which exacerbate neointimal plaque formation.

Besides regulating VSMCs, DAB2IP also modulates the stability of plaque by inhibiting pathological angiogenesis. Structurally defective newly formed vessels, promoted by vascular endothelial growth factor (VEGF) signaling around the lipid core, increase plaque vulnerability due to their high permeability and fragile nature. DAB2IP counteracts this process by binding to the VEGFR2-PI3K p85 complex, inhibiting VEGFR2-mediated pathological angiogenesis during the later phase of the VEGF response (34). An important residue (position 239) within the C2 domain of DAB2IP is essential for its interaction with vascular endothelial growth factor receptor-2 (VEGFR2) (35). DAB2IP also serves as an important regulator of inflammasome activity in pathological angiogenesis during the fibrous plaque stage. It attenuates NOD-like receptor thermal protein domain associated protein 3 (NLRP3) inflammasome-induced cytokine secretion and neovascularization by suppressing ROS production, a process mediated through its PR domain-dependent inhibition of NADPH oxidase 2 activity (36, 37). Recent studies have revealed that downregulation of DAB2IP facilitates assembly of the NLRP12-ASC-Caspase-8 inflammasome, triggering pyroptosis via N-terminal gasdermin D fragment-dependent pore formation. Vascular endothelial cells undergoing this pyroptotic process release VEGF and interleukin-1beta (IL-1β), thereby establishing a vicious cycle that promotes pathological angiogenesis (38). These findings indicate that DAB2IP is a multifaceted inhibitor of inflammasome-driven vascular pathology, working along with its function of directly inhibiting VEGFR2 to maintain plaque stability.

By modulating the phenotype of VSMCs and inhibiting pathological angiogenesis, DAB2IP coordinately protects the integrity of fibrous caps and limits neovascularization, thereby serving as a central guardian and maintaining the stability of atherosclerotic plaques at the fibrous plaque stage.

### 2.4 DAB2IP attenuates plaque destabilization and thrombosis

Plaque destabilization is characterized by thinning of the fibrous cap, expansion of the lipid core, and the accumulation of inflammatory cells and matrix metalloproteinases. These changes collectively increase susceptibility to rupture or superficial erosion with subsequent thrombus formation (20). In this critical context, DAB2IP exerts protective effects during advanced atherosclerosis by orchestrating multiple molecular mechanisms that operate by maintaining plaque stability and inhibiting the development of thrombosis.

DAB2IP may maintains the structural integrity of plaques by regulating immune processes. Studies have shown that tertiary lymphoid organs within human carotid plaque-immune cell aggregation structures are induced by chronic inflammation, and include T cells, B cells, dendritic cells, high endothelial venules, etc. (39). Given that DAB2IP has been demonstrated to effectively inhibit key pro-inflammatory signaling pathways such as NF-κB and TNF, we speculate that DAB2IP may, through its anti-inflammatory function, negatively regulate the formation and development of tertiary lymphoid organs within plaques, thereby mitigating abnormal local immune responses. Additionally, DAB2IP as a key pro-apoptotic molecule regulated by the CCR4–NOT complex during thymic positive selection, its aberrant upregulation leads to apoptosis of thymocytes at the early selection stage, thereby disrupting normal T-cell development and other crucial immunological foundations (40).

MDAB2IP maintains plaque stability by specifically regulating endoplasmic reticulum (ER) stress-induced apoptosis. When unfolded or misfolded proteins accumulate within ER, co-immunoprecipitation experiments confirm that DAB2IP interacts with IRE1α through its PH domain as verified using a PH-domain deletion mutant (DAB2IP-ΔPH), facilitating the dimerization and autophosphorylation of IRE1α. Activated IRE1α, on one hand, cleaves XBP-1 mRNA to generate the transcriptionally active spliced form, thereby enhancing the protein-folding capacity of the ER. On the other hand, DAB2IP facilitates the formation of a complex between IRE1α and TRAF2, which in turn activates the ASK1/JNK-mediated apoptotic signaling pathway. In VSMCs from DAB2IP-KO mice, the absence of DAB2IP specifically impairs the activation of the IRE1α-XBP-1/JNK signaling axis induced by ER stress, confirming that DAB2IP is required for IRE1α activation. To confirm that this regulatory mechanism is universal, the aforementioned protocol was replicated across human umbilical vein endothelial cells, bovine aortic endothelial cells, and mouse lung endothelial cells. Consistent activation patterns of DAB2IP-dependent ER stress selectivity were observed, which confirmed its conserved role in vascular endothelia (41). This protective function of DAB2IP extends to the key preservation of genomic stability, countering the instability driven by the plaque milieu.

DAB2IP serves as a key regulator of genomic homeostasis, and its loss of function may contribute to the progression of AS by compromising genomic integrity. This role is closely linked to p53 protein and DAB2IP is a key regulator of its function. DAB2IP stabilizes wild-type p53 and enhances its transcriptional activity, whereas mutant p53 binds to DAB2IP to inhibit its function (42, 43). In advanced plaques of AS, cellular senescence and persistent DNA damage are hallmarks of pathology and are closely linked to p53 signaling. For instance, activation of the p53/p21 pathway in ECs downregulates the telomere repeat binding factor 2, thereby exacerbating telomeric DNA damage and driving inflammatory responses (44). This suggests that DAB2IP may influence DNA damage repair and cellular senescence by modulating the activity and stability of p53 in AS. DAB2IP also coordinates the entire process from DNA damage response to mitotic fidelity. The deficiency of DAB2IP reduces poly ADP-ribose polymerase-1 degradation, accelerates DNA repair, and enhances G2/M checkpoint function. Furthermore, DAB2IP promotes the proper kinetochore localization and complex formation of spindle checkpoint proteins like budding uninhibited by benzimidazole-related 1 by activating polo-like kinase 1 and its downstream signaling (45). Further research has shown that cyclin-dependent kinase-phosphorylated DAB2IP enhances polo-like kinase 1 pathway activity, and inhibits the ubiquitination and degradation of cell division cycle 20 by interacting with it, thereby stabilizing the anaphase promoting complex. These mechanisms collectively ensure a robust spindle assembly checkpoint and accurate chromosome segregation (46). In summary, DAB2IP constitutes a critical molecular node connecting fundamental genomic homeostasis mechanisms with cellular senescence and DNA damage in the pathological context of atherosclerosis. However, direct evidence remains lacking, warranting further in-depth investigation.

It is noteworthy that the ultimate clinical consequence of plaque destabilization often depends on the subsequent thrombotic process. Current evidence suggests a complex role for DAB2IP in AS. Genetic studies have established that variants in the DAB2IP locus are significant determinants of plasma von Willebrand factor levels, indicating a potential systematic influence on thrombotic propensity. However, this association was not replicated in ECs, suggesting that its specific regulatory mechanism may not involve the acute secretion of von Willebrand factor from ECs (47). Furthermore, the well-documented anti-inflammatory functions of DAB2IP contribute to plaque stabilization and reduced endothelial activation, which may counterbalance its genetic effect of potentially elevating pro-thrombotic factors. Thus, the impact of DAB2IP on thrombosis is likely a composite outcome determined by the interplay between its genetic regulation of pro-thrombotic factors and its protective role in plaque stability.

## 3 DAB2IP gene inhibits coronary artery disease and cardiomyopathies

Coronary artery disease (CAD), the leading cause of cardiovascular mortality, is characterized by the formation of atherosclerotic plaques and vascular remodeling that compromise myocardial perfusion and cardiac function (48). Given the multifaceted role of DAB2IP in regulating AS, we proposed that DAB2IP may also influence cardiac diseases.

### 3.1 Multi-population genetic associations of DAB2IP with coronary artery disease and clinical implications

Genetic polymorphisms at the DAB2IP locus are closely associated with the risk of CAD and its clinical prognosis, with the effects of these associations exhibiting diversity across different populations and specific variant sites. A GWAS conducted in 2010 initially identified a single-nucleotide polymorphism (SNP), rs7025486, mapped to the *DAB2IP* gene locus at chromosome 9q33. The change in the nucleotide from G to A at this site was significantly associated with susceptibility to premature myocardial infarction (OR = 1.18, 95% CI = 1.09–1.27, *P* = 3.1 × 10^−5^) (12). The disease relevance of this locus was subsequently systematically mapped to CAD in 2012 by Harrison et al., who reported that *DAB2IP* rs7025486[A] was associated with CAD (OR = 1.16, 95% CI = 1.05–1.29, *P* = 0.003). Subsequent meta-analyses with larger sample sizes also confirmed this association (OR = 1.10, 95% CI = 1.06–1.14, *P* = 3.2 × 10^−6^) (11). Genotyping studies in Indian populations also revealed a significant association between *DAB2IP* rs7025486[A] and early-onset CAD (23). However, when the study focused on populations in East China, the independent association between *DAB2IP* rs7025486[A] and CAD was not found. This disappearance may be due to the homogeneity of the study population and the relatively low prevalence in Asian populations (24) (Table 1).

Intriguingly, other variants *of DAB2IP* also demonstrated protective effects. For example, rs587936[T], located in the intron of chromosome 9q33.2, significantly reduced the all-cause mortality risk in patients with CAD (HR = 0.65, 95% CI = 0.51–0.83, *P* < 0.050) (25). Moreover, the G allele of *DAB2IP* rs885150 was negatively associated with CAD (26). These findings collectively suggest that DAB2IP functions as a genetic modifier through pathways independent of classical risk factors (Table 1).

The negative association between SNPs of *DAB2IP* and heart diseases has also been applied in translational research. The significant association of *DAB2IP* rs7025486[A] with CAD was combined with common variants on chromosome 9p21, and the Framingham risk score improved the predictive ability for CAD (NRI = 11.1%, *P* = 0.007). In studies on Pakistani populations, the inclusion of *DAB2IP* rs7025486[A] in a genetic risk score model containing 21 variants also effectively predicted the disease (significant differences in ROC between case and control groups, *P* < 0.001) (49).

### 3.2 DAB2IP as a pivotal modulator of cardiac disease trajectories

Although several studies have revealed genetic associations between *DAB2IP* gene and CAD, its functional effect on cardiac pathobiology remains poorly defined. Transcriptomic analysis in hypoxia-exposed, urocortin II (UCN2)-treated rats indicated that UCN2 modulates a G-protein–coupled network involving DAB2IP, which appears upstream of JNK signaling. This suggests that UCN2 may exert part of its cardioprotective effect by regulating DAB2IP and influencing JNK activity, a link that remains to be experimentally confirmed (50).

Emerging evidence positions DAB2IP as a regulator of intercellular adhesion in cardiac pathology. In arrhythmogenic cardiomyopathy, plakophilin 2 primarily suppresses aberrant cell migration by maintaining intercellular adhesive structures—a function coordinated with the negative regulation of migratory pathways by DAB2IP (51). Moreover, high cell density promotes the transcription of DAB2IP, indirectly repressing YAP/TAZ signaling to fortify intercellular junctions, suggesting that DAB2IP may play a role in postinfarct repair and antifibrotic mechanisms (52).

DAB2IP may regulate primary cilia integrity in cardiac fibroblasts by stabilizing the ciliary motor protein Kinesin family member 3α (KIF3α). Primary cilia-enriched fibroblasts are prominent within the injured myocardium. KIF3α is essential for ciliary assembly and intraflagellar transport, is required for TGF-β1-mediated profibrotic responses in cardiac fibroblasts, including the synthesis of the ECM and contractile function. The depletion of KIF3α substantially attenuates TGF-β1-dependent SMAD3 phosphorylation and nuclear translocation (53). As DAB2IP stabilizes KIF3α via its N-terminal PH domain in renal epithelia, its putative role in cardiac ciliary stability and fibrosis needs to be validated, especially because its fibroblast-specific expression has not been quantified (54).

The post-menopausal predisposition to CVD originates from the disruption of the estrogen axis, wherein DAB2IP activates pathogenic cascades. Clinically, high-fat diets inhibit miR-146b-5p in ovarian granulosa cells, inducing premature ovarian failure through the activation of the DAB2IP/ASK1/p38-MAPK axis (55). This DAB2IP-driven ovarian insufficiency depletes estrogen/ERβ levels, directly amplifying cardiac TGF-β fibrotic signaling. The convergence of ovarian and cardiac mechanisms is supported by an increase in the incidence of CAD in oophorectomized women (56). Moreover, mechanisms identified in other contexts—such as the regulation of DAB2IP by estrogen receptor β (ERβ) in tumors, and the role of DAB2IP in inhibiting the ubiquitination and degradation of estrogen receptor α (ERα) in prostate cancer, suggest that DAB2IP may be involved in cardiovascular pathologies such as post-menopausal myocardial infarction and AS (57, 58). This potential connection, however, needs to be empirically established.

## 4 DAB2IP is a potential negative regulator of aortic aneurysms and aortic dissections

Aortic aneurysm (AA) is defined as irreversible dilation exceeding 1.5 times the normal vessel diameter. Moreover, AD involves endothelial rupture with the entry of blood into the false lumen, followed by paracrine or retrograde propagation, causing mesothelial/endothelial disruption and leading to insufficient perfusion of vital tissues, with Stanford type A being more common. In terms of critical cardiovascular emergencies, AA accounts for 1.3% of deaths among men who are 65–85 years old in developed nations, whereas AD presents with an incidence of approximately 3.5–5.0 per 100,000 person-years and carries an exceptionally high acute mortality, exceeding 50% within 48 h if left untreated (59, 60). The major risk determinants include hypertension, hyperlipidemia, sex, age > 65 years, active smoking, and AS (61). Although surgical intervention is the main therapeutic strategy of choice, molecular targets need to be identified for prevention, early detection, and pharmacotherapeutics.

### 4.1 Genetic architecture of DAB2IP in aortic aneurysm and dissection

Genetic evidence establishes *DAB2IP* gene is a susceptibility locus for the pathogenesis of abdominal aortic aneurysm (AAA). A GWAS revealed that the *DAB2IP* rs7025486[A] variant (chr9q33) confers the risk of AAA in European and American populations (OR = 1.21, 95% CI = 1.11–1.32, *P* = 4.6 × 10^−10^) [(12)]. Subsequent transcriptional profiling revealed a 2.29-fold increase in the expression of DAB2IP in AAA tissues, although this increase was statistically underpowered due to the limited number of samples (*n* = 18 cases/15 controls) (62). Validation in a contemporary multicenter cohort (2016–2019; 148 AAA patients vs. 50 controls) confirmed significant *DAB2IP* rs7025486[A] enrichment in AAA patients (*P* = 0.040), with the results of a haplotype analysis demonstrating a high frequency of minor alleles (25.5%; *P* = 0.028). Among the rs7025486[A] carriers who developed aneurysms, those with a cross-sectional morphology with a diameter ≥50 mm predominated, whereas those with an anatomic distribution had no preference for a specific site. Further differential analysis revealed that this association was amplified in hypertensive AAA patients (OR = 3.30, 95% CI = 1.70–6.37, *P* < 0.001) (22), and was strongly correlated with aneurysm dilation in females (*P* ≤ 0.020) (Table 1).

However, evidence linking the *DAB2IP* gene to AD remains preliminary. A Chinese whole-exome sequencing study of 99 sporadic AD patients conducted in 2020 identified the *DAB2IP* gene as a candidate susceptibility gene (63), but the findings need to be validated in ethnically diverse cohorts with expanded sample sizes.

Apart from the genetic associations outlined above, the mechanistic links between DAB2IP and aortic pathologies remain exploratory. As DAB2IP plays a crucial role in mitigating atherosclerosis, a key risk factor for AA/AD, DAB2IP may protect against aortic diseases by suppressing shared pathological pathways, such as those driven by hemodynamic stress and metabolic dysregulation.

## 5 DAB2IP in cardiovascular disease: broader implications and therapeutic prospects

Early studies suggested a potential role for DAB2IP in hemodynamic homeostasis. The data obtained from a GWAS first identified SNPs in DAB2IP (rs35061590 and rs13290547) as modulators of heart rate (*β* = 16.03, *P* = 9.5 × 10^−6^) (64). Subsequent meta-analysis showed significant positive correlations between *DAB2IP* cg13696706 methylation levels and both systole (SBP: *P* = 9.8 × 10-8, DBP: *P* = 0.001; FDR < 0.05). However, the results of the bivariate structural equation model analysis revealed that the contributions of genetic and environmental factors to the correlation between cg13696706 methylation and SBP are not significant, suggesting that this association may be driven mainly by other factors. Further studies are needed to clarify these factors (65). More recently, genome-wide methylation confirmed that the methylation level of *DAB2IP* is positively correlated with DBP (SLK-*P* = 0.030), albeit with a high false discovery rate (66). Given the early establishment of the association of DAB2IP with hemodynamic parameters and the subsequent lack of mechanistic breakthroughs or conclusive findings, the available evidence remains predominantly correlative. Therefore, we have presented these associations here to emphasize this scientific gap and encourage future investigations into the underlying mechanisms.

While males have a 3–6-fold greater incidence of AAAs, a critical epidemiological paradox emerges in female patients who show accelerated disease progression (0.41 mm/year greater growth velocity) and a high risk of rupture despite a lower prevalence. This sex-specific disparity has been mechanistically linked to the *DAB2IP* rs7025486[A] polymorphism, where a stronger disease association is observed in women—a phenomenon amplified under high pulse pressure conditions after adjustment for hemodynamic confounders (63).

Some researchers hypothesized that DAB2IP, a known modulator of androgen signaling in oncology, may also interact with sex hormone pathways in the cardiovascular system. Specifically, DAB2IP may influence vascular remodeling and arterial stability by modulating androgen receptor (AR) activity and its downstream signaling pathways, thereby playing an important role in CVD. When DAB2IP levels are low, the PI3K/AKT/mTOR/ETS1 axis is activated, which in turn increases the AKR1C3-mediated conversion of dehydroepiandrosterone to testosterone and reactivates AR signaling post-castration (67). Additionally, DAB2IP can inhibit AR-mediated pathways in an androgen-independent manner, such as by inhibiting the nuclear translocation and phosphorylation of AR or by competitively binding to Src to reduce its activity. In turn, AR signaling suppresses the expression of DAB2IP, thereby establishing a positive feedback loop (68, 69)**.** Besides having regulatory effects on AR, the loss of DAB2IP also stabilizes the mitochondrial transmembrane potential, preventing the release of cytochrome c, Omi/HtrA2, and Smac from mitochondria to the cytoplasm, thereby directly counteracting the apoptosis induced by androgen deprivation therapy (70).

This hypothesis is supported by several lines of evidence: First, PPI analysis revealed an interaction between DAB2IP and the AR in patients with AD (13). Second, clinical observations of greater cardiovascular risk in patients receiving androgen deprivation therapy further suggest that androgen signaling pathways are important for maintaining cardiovascular homeostasis (71).

DAB2IP offers a promising framework for developing precision medicine, guiding stage-specific interventions to decrease cardiovascular risk in diabetic patients. Under early high-glucose conditions, the expression of DAB2IP is upregulated, which subsequently inhibits the HIF-1α/VEGF pathway and impairs vascular function. In contrast, high insulin levels partially reverse this glucose-induced upregulation of DAB2IP (34). However, in the advanced clinical stage, the expression of DAB2IP is strongly negatively correlated with DAB2IP is strongly negatively correlated with obesity-related type 2 diabetes indices (HOMA-IR and WHR). Consistently, low DAB2IP levels have been detected in adipose tissue from obese patients with type 2 diabetes (72). DAB2IP deficiency is associated with the activation of the JNK/p38-MAPK/ERK1/2 signaling cascade, an increase in TNF-α and C-reactive protein levels, and further exacerbates insulin resistance. This biphasic pattern suggests that early increase in DAB2IP levels may increase cardiovascular risk, while its late-stage downregulation promotes cardiovascular risk through aggravated insulin resistance. These findings suggest that DAB2IP can serve as a potential early predictive biomarker for obesity-related type 2 diabetes. Early interventions targeting glycemic control may help reduce DAB2IP-mediated vascular damage. In advanced stages, therapeutic strategies aimed at modulating the expression of DAB2IP and its downstream pathways can mitigate insulin resistance-induced cardiovascular complications.

Targeting DAB2IP through epigenetic regulatory mechanisms may mitigate AAA. The results of qPCR analysis demonstrated that the expression of EZH2 and miR-363-3p was downregulated by about 3.28-fold and 3.62-fold, respectively, in the AAA group. The results of Pearson correlation coefficient analysis revealed moderate negative correlations between DAB2IP and both EZH2 (R = −0.45) and miR-363-3p (R = −0.40), suggesting that EZH2 and miR-363-3p exert limited negative regulatory effects on DAB2IP. Although EZH2 inhibitors have been explored in CVD, such as suppressing the proliferation of VSMCs in AS and vascular restenosis (73), no agents have yet been approved for clinical practice in CVD. The miR-363-3p firstly serves as a negative regulator of DAB2IP in AAA, though lacking detailed mechanistic insights and requiring further experimental validation (62).

Some researchers have identified circDAB2IP as a potential plasma biomarker for the dysfunction of pediatric organs following congenital heart surgery. Its levels are significantly downregulated during postoperative organ dysfunction (74). We proposed a mechanistic hypothesis, which stated that circDAB2IP may function as a “molecular sponge”, sequestering negative regulators, which can indirectly alleviate the epigenetic suppression of its host gene, *DAB2IP*. This action may synergize with the inherent anti-inflammatory function of DAB2IP to maintain cardiovascular homeostasis. Consequently, the postoperative decrease in circDAB2IP can attenuate this protective mechanism, potentially exacerbating the inflammatory state driven by DAB2IP deficiency. Thus, the emergency delivery of circDAB2IP via engineered exosomes is a suitable theoretical strategy to mitigate the “inflammatory storm” associated with postoperative organ dysfunction. However, this approach faces substantial challenges, including low targeting specificity of exosomes, high heterogeneity of exosomes, and significant manufacturing hurdles. Overcoming these technical barriers, for example, by engineering exosomes with targeting peptides for enhanced specificity, using antisense oligonucleotides for precise circRNA engineering, and refining production processes to mitigate risks, is important for advancing their clinical feasibility (75). Ultimately, the definitive role and therapeutic potential of circDAB2IP in cardiovascular diseases need to be confirmed through more rigorous experimentation.

## 6 DAB2IP in cardiovascular brain crosstalk regulation

**Figure 2:**
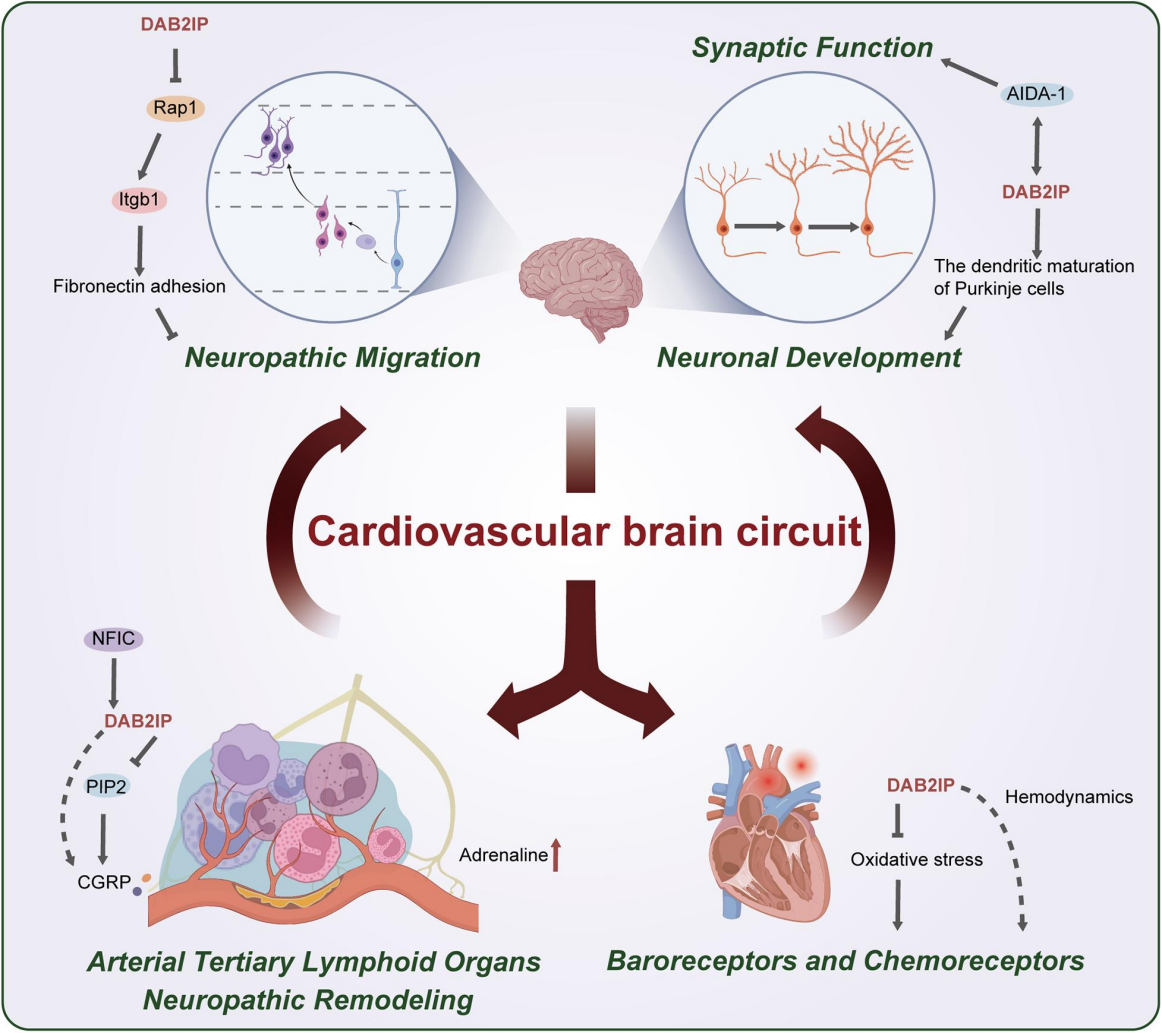
Roles of DAB2IP in the cardiovascular brain circuit. (1) Tertiary lymphoid organs form around atherosclerotic plaques, a process in which DAB2IP-mediated immune cell recruitment may be involved; (2) Upregulation of DAB2IP expression by NFIC may modulate the release of CGRP; (3) Impaired PIP2-mediated calcium release resulting from DAB2IP deficiency may lead to aberrant CGRP release; (4) The positive correlation between DAB2IP and plasma epinephrine levels may partially reflect sympathetic nerve remodeling at vascular lesion sites, which contributes to increased epinephrine; (5) DAB2IP suppress oxidative stress, which could stimulate chemoreceptors. The association of DAB2IP with blood pressure and heart rate suggests its potential role in modulating baroreceptor activity; (6) Loss of DAB2IP upregulates Rap1 activity, stimulating integrin β1-mediated fibronectin adhesion and thereby inhibiting neuronal migration; (7) DAB2IP promotes dendritic maturation in cerebellar Purkinje cells; (8) The high binding affinity between DAB2IP and AIDA-1 indicates its potential role in regulating synaptic function.

The cardiovascular brain crosstalk (CBC) comprises the well-studied HBC and the emerging ABC, integrating bidirectional neural pathways required for systemic homeostasis. Some studies have reported that DAB2IP is a key modulator of CBC plasticity, particularly in response to vascular inflammation and neural remodeling (Figure 2).

### 6.1 DAB2IP in artery-brain circuit

In CVD, pathological neural remodeling can lead to aberrant signaling in the ABC. This is characterized by the reorganization of sensory nerves (which form the ABC “sensors”) and sympathetic nerves (which constitute the ABC “effectors”) within the adventitia of the abdominal aorta. This reorganization is an adaptive response of the peripheral nervous system to the inflammatory microenvironment mediated by immune cells (1).

The sensory mechanism of the ABC involves various inflammatory mediators and neuropeptides, such as calcitonin gene-related peptide (CGRP). These molecules activate nociceptive receptors (e.g., transient receptor potential vanilloid 1) on sensory nerve axon terminals, triggering action potentials. When DAB2IP levels are inadequate, enhanced immune cell recruitment promotes the formation of arterial tertiary lymphoid organs (39) and the release of abundant inflammatory mediators. Moreover, the deficiency of DAB2IP may increase PIP2 levels dependent on the loss of its Arf6 GAP activity (17), may promote the PLC-IP₃-calcium release pathway, and enhance neuronal excitability and CGRP release. Ultimately, it synergizes with the inflammatory milieu to amplify nociceptive signaling (76). In addition, studies have found that the transcription factor NFIC directly binds to the promoter region of the *DAB2IP* gene and positively regulates its transcription (77). Meanwhile, NFIC is also involved in modulating the expression of CGRP in DRG neurons, which are responsible for nociceptive signaling from deep tissues (78). In a chronic stress-induced model of visceral hypersensitivity, a significant downregulation of DAB2IP expression has been observed (79). Based on these findings, it is hypothesized that DAB2IP may act as a downstream effector of NFIC, participating in the regulation of CGRP and other pain-related mediators released from DRG neurons.

These action potentials are transmitted via the dorsal root ganglion (DRG) to the central nervous system, where they are integrated in brainstem nuclei before regulating vascular adventitial function through sympathetic efferent pathways. An increase in adrenaline levels in regions of pathological neural remodeling serves as direct evidence for the overactivation of the sympathetic nervous system. Some studies have shown a correlation between DAB2IP and plasma adrenaline levels, which may partially reflect the imbalance in sympathetic regulation resulting from the effect of DAB2IP deficiency on ABC signaling (80). DAB2IP may also indirectly suppress aberrant neural remodeling by inhibiting pathological angiogenesis and maintaining vascular microenvironment homeostasis.

Although cerebrovascular disease can directly damage ABC structures, early case-control studies limited by small sample sizes did not find significant associations between the rs7025486[A] variant and cerebrovascular pathologies (intracranial aneurysms, ischemic stroke, or its large-artery and cardioembolic subtypes). The statistical power of these studies was insufficient to detect variants with small effect sizes (12) (Table 1).

### 6.2 DAB2IP in heart-brain circuit

The heart-brain circuit (HBC) is a multilayer communication system whose core architecture comprises a sensory arm (vagus and spinal nerves) that receives cardiac signals, brainstem centers for integrating information, extrinsic autonomic (sympathetic and parasympathetic) efferents for rapid regulation, and the intrinsic cardiac nervous system responsible for local fine-tuning (1). In this section, we discussed the potential role of DAB2IP in sensory conduction within the HBC and comprehensively analyzed how DAB2IP-mediated regulation of the cerebral cortex, a high-level integration center, exerts top-down fine-tuning of cardiac function.

Cardiac signals are detected by specialized receptors and transmitted via the vagus and spinal nerves. Among these, the baroreceptors (sensing blood pressure changes) and chemoreceptors (sensing blood oxygen/carbon dioxide levels) located in the aortic arch are very important. Based on the known roles of DAB2IP in maintaining vascular integrity and compliance, as well as its association with blood pressure and heart rate, we speculated that DAB2IP may modulate the encoding of hemodynamic signals by baroreceptors. Moreover, as DAB2IP is a key negative regulator of oxidative stress, DAB2IP deficiency exacerbates HIF-1α-mediated local tissue hypoxia. We further hypothesized that this hypoxic state may increase the sensitivity of chemoreceptors to low-oxygen signals, thereby promoting the transmission of abnormal signals to higher central nuclei.

Cardiac interoceptive signals are ultimately integrated and processed in higher centers. Studies have found that the ABC and the HBC share certain key brain regions, such as the insula (closely related to emotion and interoception). DAB2IP coordinates cortical neuronal migration during brain development. It is highly expressed in the adult cortex, with its 110-kDa isoform enriched in axons and the intermediate zone on embryonic day 14, peaking in the cortical plate by embryonic day 16. Downregulation of DAB2IP leads to the arrest of the migration of late-born neurons, primarily due to failed multipolar-to-bipolar transition, causing neurons to stagnate in the intermediate zone. The expression of DAB2IP requires precise regulation—its deficiency impedes migration, whereas its overexpression induces neuronal death and disrupts signaling (81). Mechanistically, DAB2IP, as a dual-specificity GAP, operates independently of Reelin signaling, depending on its subcellular localization (governed by the PH/C2 domains) determines Ras/Rap1 specificity. In *DAB2IP* knockdown brains, activated Rap1, but not Ras, increases significantly, enhancing β1-integrin-mediated fibronectin adhesion. This mislocalizes late-born neurons (e.g., C(ux1^+^ layers II-IV; Brn-2^+^ layers II-III) to deeper cortical zones (82).

DAB2IP is also required for maintaining neuronal morphology. Cortical neurons lacking DAB2IP develop shorter neurites with higher branching points, which is functionally supported by its role in maintaining microtubule-associated proteins such as MAP2, MAP1b, and Tau (81, 83). Given that the expression of DAB2IP is the highest in the cerebellum among brain regions, we further investigated its role in cerebellar neuronal formation to comprehensively understand its mechanism of action. DAB2IP regulates the dendritic maturation of Purkinje cells; its knockout delays dendritic arborization, reduces distal parallel fiber synapses, and increases proximal climbing fiber synapses. DAB2IP does not co-localize with the glutamate receptor δ2, suggesting that it does not directly participate in postsynaptic signaling (84). However, DAB2IP may bind with high affinity (Kd = 70 nM) via its NPVY motif to the PTB domain of the postsynaptic scaffold protein AIDA-1, thereby indirectly modulating synaptic structure and function (85), although its *in vivo* functional relevance needs to be validated. Moreover, the structural intersection between DAB2IP and DAB2, a known inhibitor of neurite extension via the RhoA/ROCK pathway, suggests that the function of DAB2IP may involve the regulation of DAB2-mediated neurite outgrowth (86).

Synthesizing these findings, we proposed a testable hypothesis, which stated that if the regulatory roles of DAB2IP in neuronal migration and morphogenesis extend to subcortical centers of the cardio-brain circuit, such as the dorsal motor nucleus of the vagus nerve and the nucleus tractus solitarius, then its functional deficiency could disrupt precise neuronal assembly and signal integration within these central CBC components through mechanisms of neuronal misplacement, impaired dendritic maturation, and aberrant synaptic connectivity.

Although the neuroimmune theory elucidates mechanisms by which CBC directly mediates neuroimmune circuits via factors such as Netrin-1, promoting macrophage migration out of plaques (87), the mechanism of DAB2IP of action is quite different. It is not expressed in macrophages and does not affect their total numbers; instead, it participates in the recruitment of immune cells by acting on endothelial cells to regulate chemokine release. This finding indicates that DAB2IP does not directly mediate typical neuroimmune crosstalk but rather provides foundational support for the higher-order regulation of the CBC by shaping the structural and functional integrity of the neural circuit.

### 6.3 DAB2IP-mediated neuro-cardiovascular crosstalk

**Figure 3:**
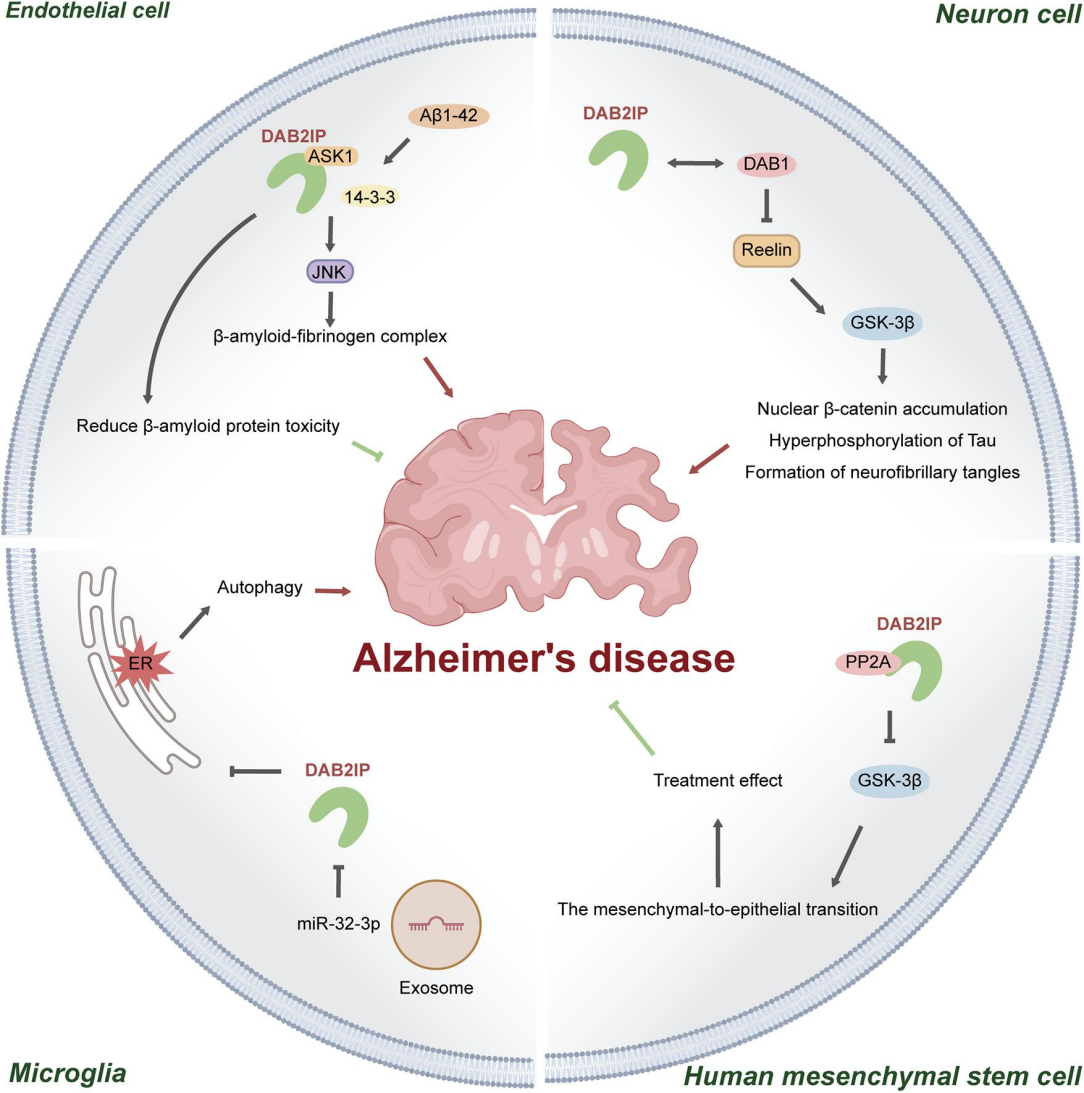
Mechanisms of DAB2IP in Alzheimer's disease. (1) In cerebrovascular endothelial cells, amyloid-β₁–₄₂ promotes DAB2IP-mediated activation of the ASK1–JNK apoptosis signaling pathway. Conversely, DAB2IP itself alleviates amyloid-β toxicity; (2) DAB2IP interacts with DAB1 and may potentiate Reelin-induced activation of GSK-3β, thereby exacerbating disease progression; (3) In hMSCs, DAB2IP forms a complex with PP2A, inhibiting GSK-3β–mediated mesenchymal–epithelial transition and contributing to the reparative function of hMSCs; (4) Exosomes derived from hMSCs deliver miR-32-3p, which downregulates DAB2IP expression, promotes ER stress–induced microglial autophagy, and attenuates the therapeutic efficacy of hMSCs.

DAB2IP is involved in neurodegenerative diseases, most notably Alzheimer's disease (Figure 3). Alzheimer's disease is a neurodegenerative disorder originating in the hippocampus, characterised by amyloid plaques and neurofibrillary tangles (88, 89).

Genetic studies have confirmed the association between the *DAB2IP* gene and Alzheimer's disease. Cross-trait meta-analysis revealed that the G allele of *DAB2IP* rs10818576 is positively associated with Alzheimer's disease (27) (Table 1). Paradoxically, the DAB2IP gene is hypermethylated in the dorsolateral prefrontal cortex of patients with Alzheimer's disease and major depressive disorder compared to controls (ΔBeta = 0.03, *P* = 0.001; ΔBeta = 0.004, *P* = 6.0 × 10^−3^) (90) (Table 2). The hippocampus, as an early-affected region, may show compensatory upregulation of DAB2IP; the dorsolateral prefrontal cortex, as a late-affected region, enters a state of pathological decompensation marked by its downregulation.

**Table 2:** Association of DAB2IP differential methylation with nervous diseases.

Gene (elements)	Phenotype	ΔBeta	*p*-value	Ref.
*DAB2IP (Body; Island)*	Alzheimer's disease	0.03	0.001	(90)
*DAB2IP (Body; N_Shore)*	MDD	0.004	0.006	(90)
*DAB2IP* cg14021373	DEACMP	1.54	0.004	(91)

Genomic regions of DNA methylation alteration include gene body, CpG islands, northern/southern shores (regions up to 2 kb from CpG island, upstream and downstream), northern/southern shelves (regions from 2 to 4 kb from CpG island, upstream and downstream), and open sea (the rest of the genome).

DEACMP, encephalopathy after carbon monoxide poisoning; HR, hazard ratio; MDD, major depressive disorder; OR, odds ratio; ΔBeta, coefficient for differential methylation between disease and control samples.

A transcriptome-wide association study of hippocampal neurons conducted in 2019 revealed a 7.52-fold upregulation of DAB2IP at the transcript level and a 5.23-fold upregulation at the gene level in Alzheimer's patients compared to controls (92). Studies have further elucidated the disease-stage heterogeneity of the expression of DAB2IP. The *DAB2IP* gene showed lower expression in the C1 subgroup with relatively higher CDK5R1 expression but higher expression in the C2 subgroup with relatively lower CDK5R1 expression (93). This expression pattern suggests that DAB2IP focuses on more neuronal dysfunction directly resulting from Reelin signaling disruption, compared with immunoinflammatory regulation. Furthermore, DAB2IP interacts with the adaptor protein DAB1 in the Reelin pathway (89). We hypothesised that DAB2IP dysfunction may disrupt Reelin signaling, leading to aberrant activation of GSK-3β, which in turn could promote nuclear β-catenin accumulation, hyperphosphorylation of Tau, and the formation of neurofibrillary tangles. These disturbances may contribute to a vicious cycle linking Aβ and Tau pathology, ultimately exacerbating synaptic dysfunction and neuroinflammation, the key pathological hallmarks of Alzheimer's disease (94).

DAB2IP also regulates the dysfunction of the BBB. In Alzheimer's disease, β-amyloid is deposited on cerebrovascular surfaces, contributing to cerebral amyloid angiopathy, while cerebral endothelial cells, as the primary component of the BBB, play protective roles (89). Mechanistically, β-amyloid-1-42 significantly upregulates the expression of DAB2IP in brain cerebral microvascular endothelial cells, promoting the activation of ASK1-JNK/p53 signaling via 14-3-3 dissociation to induce CEC apoptosis (95). This apoptotic cascade compromises the integrity of the BBB, facilitating the formation of the β-amyloid-fibrinogen complex to aggravate Alzheimer's disease. At the same time, Aβ produced in the brain can cross the dysfunctional BBB, enter the systemic circulation, and be deposited in the heart, potentially leading to cardiac dysfunction or even heart failure (96). However, the upregulation of DAB2IP by β-amyloid protein also depends on the predicted farnesylation site to reduce β-amyloid protein toxicity (97). Furthermore, NF1 is predicted by TRRUST v2 analysis to upregulate DAB2IP in Alzheimer's disease, engaging pathways implicated in endothelial function and vascular morphogenesis, which are key processes for the BBB. This prediction, however, requires experimental confirmation (92).

Moreover, DAB2IP regulates the therapeutic effects of human mesenchymal stem cells (hMSCs) in Alzheimer's disease. In hMSCs, the C2 domain-dependent PP2A–DAB2IP complex suppresses the activity of GSK-3β by reducing its phosphorylation at the Ser9 site (98). Thus, maintaining the expression of DAB2IP in hMSCs helps prevent excessive activation of GSK-3β in mature neurons, thereby mitigating damage to newly generated neurons. However, the suppression of GSK-3β activity also inhibits the mesenchymal-to-epithelial transition, which may compromise the therapeutic potential of hMSCs in Alzheimer's disease. Moreover, the transplantation of MSCs can induce microglial autophagy, whose dysfunction is a recognized pathogenic factor in Alzheimer's disease. Exosomal miR-32-3p derived from MSCs targets the 3′-UTR of DAB2IP, downregulating its expression and triggering ER stress-initiated autophagy in microglia (99). High levels of DAB2IP prevent the differentiation of hMSCs into neuronal epithelial cells, and may simultaneously enhance miR-32-3p production. The increased miRNA suppresses DAB2IP in microglia, which in turn exacerbates autophagy and reduces inflammatory factor release (96, 100).

In summary, DAB2IP regulates multiple pathways within the brains of Alzheimer's disease patients that may promote CBC. The clinical association between Alzheimer's disease and cardiovascular autonomic dysfunction further suggests a potential key role for DAB2IP in regulating neuro-cardiovascular crosstalk related to Alzheimer's disease (100).

## 7 Discussion

Originally characterized as a tumor suppressor, DAB2IP was later found to act as a pleiotropic regulator central to cardiovascular and neurological pathophysiology. This gene encodes a protein featuring a conserved multidomain scaffolding architecture (PH, C2, GAP, and PER domains), enabling integrative modulation of oxidative stress responses, immunoinflammatory cascades, and cellular processes, including spanning migration, proliferation, and apoptosis.

DAB2IP exhibits notable anti-inflammatory effects using distinct molecular mechanisms to achieve a common goal, with its function being highly dependent on the cellular context. In ECs, it alleviates inflammation by preventing the assembly of the TLR4-MyD88 complex and inhibiting TNF-α-mediated activation of NF-κB. In VSMCs, it suppresses the IFN-γ-driven JAK2/STAT pathway, thereby reducing the release of pro-inflammatory chemokines. DAB2IP also functions as a “bidirectional regulator” in maintaining vascular homeostasis, with its activity displaying a temporal dimension. During the early stages of disease, the induction of endothelial apoptosis—mediated through the activation of the ASK1-JNK/p38 pathway and internalized TNFR2 signaling—may serve as a quality control mechanism to eliminate damaged cells in response to initial injury. In advanced stages, DAB2IP stabilizes the contractile phenotype of VSMCs by inhibiting the abnormal activation and migration of these cells, thereby arresting the progression of plaques. The spatiotemporal specificity of DAB2IP is a major challenge for targeted therapy: future interventions must move beyond systemic agonism or inhibition and achieve cell-specific drug delivery. Otherwise, benefits gained in one process may be offset by adverse effects elicited in another process.

Population-based studies have demonstrated robust associations between the SNPs of DAB2IP and cardiocerebrovascular disorders, including AS, AA, CAD, and Alzheimer's disease, with notable population-specific susceptibility and sex-dimorphic predisposition. DAB2IP is a critical regulator of CBC plasticity, modulating both ABC (suppressing vascular inflammation and pathological neural rewiring) and HBC (regulating cardiovascular function and neural processes). DAB2IP dysregulation links the pathology of Alzheimer's disease (via BBB dysfunction and systemic inflammation) to the risk of CVD and influences neural repair. However, the precise pathways underlying DAB2IP-mediated bidirectional neuro-cardiovascular communication remain a key gap requiring future mechanistic elucidation.

## 8 Conclusion

DAB2IP is an important bridging oncology, cardiovascular biology, and neuroscience. It is a promising therapeutic target for precision medicine. Its scaffold-dependent signaling modulation offers a novel paradigm for intercepting shared pathways in cardiocerebral comorbidities.
